# Real-time prostate motion assessment: image-guidance and the temporal dependence of intra-fraction motion

**DOI:** 10.1186/1756-6649-13-4

**Published:** 2013-09-23

**Authors:** Avilash K Cramer, Amanu G Haile, Sanja Ognjenovic, Tulsee S Doshi, William Matthew Reilly, Katherine E Rubinstein, Nima Nabavizadeh, Thuan Nguyen, Lu Z Meng, Martin Fuss, James A Tanyi, Arthur Y Hung

**Affiliations:** 1 Brown University, Providence, RI, USA; 2 Oregon State University, Corvallis, OR, USA; 3 Stanford University, Stanford, CA, USA; 4 Pomona College, Claremont, CA, USA; 5 Whitman College, Walla Walla, WA, USA; 6 Department of Radiation Medicine, Oregon Health & Science University, Portland, OR, USA; 7 Department of Public Health & Preventive Medicine, Oregon Health & Science University, Portland, OR, USA; 8 Department of Radiation Oncology, University of California Davis Comprehensive Cancer Centre, Sacramento, California, USA; 9 Department of Nuclear Engineering & Radiation Health Physics, Oregon State University, Corvallis, OR, USA

**Keywords:** Prostate cancer, Real-time motion tracking, Intrafraction variation, Treatment margin, Treatment time

## Abstract

**Background:**

The rapid adoption of image-guidance in prostate intensity-modulated radiotherapy (IMRT) results in longer treatment times, which may result in larger intrafraction motion, thereby negating the advantage of image-guidance. This study aims to qualify and quantify the contribution of image-guidance to the temporal dependence of intrafraction motion during prostate IMRT.

**Methods:**

One-hundred and forty-three patients who underwent conventional IMRT (n=67) or intensity-modulated arc therapy (IMAT/RapidArc, n=76) for localized prostate cancer were evaluated. Intrafraction motion assessment was based on continuous RL (lateral), SI (longitudinal), and AP (vertical) positional detection of electromagnetic transponders at 10 Hz. Daily motion amplitudes were reported as session mean, median, and root-mean-square (RMS) displacements. Temporal effect was evaluated by categorizing treatment sessions into 4 different classes: IMRT_c_ (transponder only localization), IMRT_cc_ (transponder + CBCT localization), IMAT_c_ (transponder only localization), or IMAT_cc_ (transponder + CBCT localization).

**Results:**

Mean/median session times were 4.15/3.99 min (IMAT_c_), 12.74/12.19 min (IMAT_cc_), 5.99/5.77 min (IMRT_c_), and 12.98/12.39 min (IMRT_cc_), with significant pair-wise difference (p<0.0001) between all category combinations except for IMRT_cc_ vs. IMAT_cc_ (p>0.05). Median intrafraction motion difference between CBCT and non-CBCT categories strongly correlated with time for RMS (t-value=17.29; p<0.0001), SI (t-value=−4.25; p<0.0001), and AP (t-value=2.76; p<0.0066), with a weak correlation for RL (t-value=1.67; p=0.0971). Treatment time reduction with non-CBCT treatment categories showed reductions in the observed intrafraction motion: systematic error (Σ)<0.6 mm and random error (σ)<1.2 mm compared with ≤0.8 mm and <1.6 mm, respectively, for CBCT-involved treatment categories.

**Conclusions:**

For treatment durations >4-6 minutes, and without any intrafraction motion mitigation protocol in place, patient repositioning is recommended, with at least the acquisition of the lateral component of an orthogonal image pair in the absence of volumetric imaging.

## Background

Intensity-modulated radiotherapy (IMRT) techniques for prostate cancer facilitate safe dose escalation through maximization of the therapeutic ratio with proven benefits on local recurrence and biochemical control rates [1,2]. Given the very conformal nature of IMRT treatment plans, accurate patient positioning utilizing image-guidance systems is of the utmost importance [3]. Commercial availability of real-time or near-real-time monitoring devices [4-7] has permitted detailed evaluation of intrafraction prostate motion. Consequentially, it is now known that for a typical radiotherapy fraction, motion is largest in the antero-posterior and cranio-caudal axes [8,9], and that the probability of intrafraction motion has a temporal dependence [5,10,11]. The latter finding is vital, as IMRT treatment times are typically longer than their conventional counterparts.

Curtis *et al*. [7] recently showed that with volumetric image-guidance every 4 minutes, a 3-mm margin is sufficient to account for intrafraction motion errors in the absence of positional correction measures based on real-time continuous tracking of the prostate. Shelton *et al*. [6], however, showed that an optimized workflow with faster treatment techniques, such as intensity-modulated arc therapy (IMAT), allows for a significant decrease in prostate intrafraction motion errors. Therefore, the current study aims to evaluate the temporal dependence of intrafraction motion of the prostate gland on image-guided IMRT techniques in the absence of positional correction measures based on real-time continuous tracking of the prostate. We hypothesize that the addition of image-guidance on prostate IMRT results in significantly longer treatment times, resulting in significant intrafraction motion error.

### Methods

#### Patient population and treatment planning

The current retrospective analysis was approved by our Institutional Review Board (IRB), with patient informed consent waiver. Medical records of patients that underwent prostate IMRT or IMAT between September 2007 and July 2011 were reviewed. A cohort of 143 patients (mean/median age: 68/67; range: 51–87) with histologically confirmed clinical stage I-III prostate adenocarcinoma formed the basis of the current analysis.

Simulation and radiotherapy planning techniques have been reported previously [12]. In brief, simulation computed tomography (CT) images were acquired in the treatment position with the patient supine and transferred to a three-dimensional dosimetric planning platform (Pinnacle^3^ v7.6, Philips Medical Systems, Andover, MA or Eclipse v8.6/8.9, Varian Medical Systems, Inc., Palo Alto, CA, USA) for structure segmentation and treatment planning. Seven to 9-field step-and-shoot or sliding window IMRT plans (n=67) were computed for a prescribed dose of 70 Gy (2.5 Gy/28 fractions) or 78 Gy (2 Gy/39 fractions). Two sequential-arc (358° each) IMAT plans (n=76) were computed for a prescribed dose of 70 Gy (2.5 Gy/28 fractions).

#### Daily target localization and intrafraction motion analysis

Patient setup procedures have also been previously described [12]. In brief, daily localization was based on electromagnetic transponder detection, validated at least weekly by volumetric cone-beam CT (CBCT; Varian Medical Systems, Palo Alto, CA, USA). Intrafraction motion of the prostate gland was monitored in real-time with 1/10^th^ s update of the lateral (LR/X), vertical (AP/Y), and longitudinal (SI/Z) displacement of the localized target isocentre. Sustained deviations >4 mm in any translational direction prompted corrective action while transient deviations lasting <1 second were ignored. Tracking data points, obtained immediately post electromagnetic transponder localization and up to 30 s after dose administration, were used for the current evaluation. Tracking data with unusually large displacement of the target isocentre at the terminal portion of the tracking session (due to motion of the patient support assembly or treatment couch prior to discontinuation of real-time tracking) were excluded. Records of treatment fractions that were corrupted (i.e., containing no tracking data) were also excluded.

#### Intrafraction positional error estimation

Tracking sessions were separated into 4 distinct “treatment categories:” IMRT_c_ (n=1226; IMRT sessions with setup based on electromagnetic transponders only), IMRT_cc_ (n=864; IMRT sessions with setup based on electromagnetic transponders plus CBCT verification), IMAT_c_ (n=1461; IMAT sessions with setup based on electromagnetic transponders only) and IMAT_cc_ (n=586; IMAT sessions with setup based on electromagnetic transponders plus CBCT verification). Digital tracking data and associated time stamps were downloaded to Microsoft Excel (2007; Microsoft, Redmond, WA, USA) for processing. The root-mean-square (RMS) distance was calculated for each time point.

For reporting the individual directional components of target motion, the absolute values of motion displacements were used. For the purpose of this study, +X, +Y, and +Z coordinates defined displacement to the left, posterior, and superior directions from isocentre, respectively. Daily directional components were synchronized at each time point, and the population-based average computed for each treatment category was used to evaluate motion trend over time. Daily treatment couch corrections for sustained deviations >4 mm were reported.

To summarize the large amount of data points for each daily session into an analyzable endpoint for the entire cohort, the median observed absolute displacement was used. Furthermore, for each treatment category, population-based mean (M), systematic (Σ), and random (σ) errors were computed for each motion direction. Population-based effect of treatment duration on intrafraction motion amplitude was also assessed by computing displacement probabilities as a function of displacement and time.

#### Statistical analysis

Correlations between outcome (dependent) variable and effect variables were analyzed using a linear mixed effects model. The primary outcome of interest was intrafraction motion (median LR, median SI, median AP, and median RMS) and the measured covariate was session treatment time. Subject specificity served as random effect while the fixed (main) effect was the treatment category (IMRT_c_, IMRT_cc_, IMAT_c_, and IMAT_cc_). Heterogeneity between treatment categories was taken into account. Hypothesis testing for all pair-wise comparisons were adjusted for the family-wise errors across multiple comparisons at the 0.05 level using the Bonferroni correction. All statistical analyses were performed using SAS statistical software (version 9.0; SAS Inc, Cary, NC, USA).

## Results

### Magnitude and duration of prostate displacement

**Figure 1:**
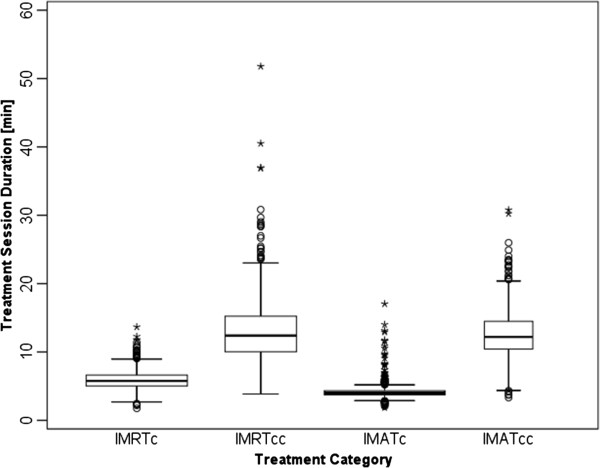
**Box-and-whisker plots of the distribution of treatment session times for each treatment category.** Whiskers denote the nearest values <1.5 times the interquartile range (IQR), open circles beyond the whisker lines (^o^) indicate individual outliers (<3.0 times the IQR), and stars (*) signify extreme values (>3.0 times the IQR).

Figure 1 summarizes the duration of treatment sessions per treatment category. The observed mean/median session duration were 4.15/3.99 min (IMAT_c_), 5.99/5.77 min (IMRT_c_), 12.74/12.19 min (IMAT_cc_), and 12.98/12.38 min (IMRT_cc_); overall range: 1.8 min (IMRT_c_) to 51.8 min (IMRT_cc_). Pair-wise comparison between treatment categories showed significant differences (p<0.0001), except for IMRT_cc_ vs. IMAT_cc_ (p>0.05). Although not the original intent of the current study, a comparison between CBCT (least square mean = 12.88 min) and non-CBCT (least square mean = 4.99 min) categories also showed a statistically significant difference (least square mean difference = 7.90 min; p<0.0001).

**Figure 2:**
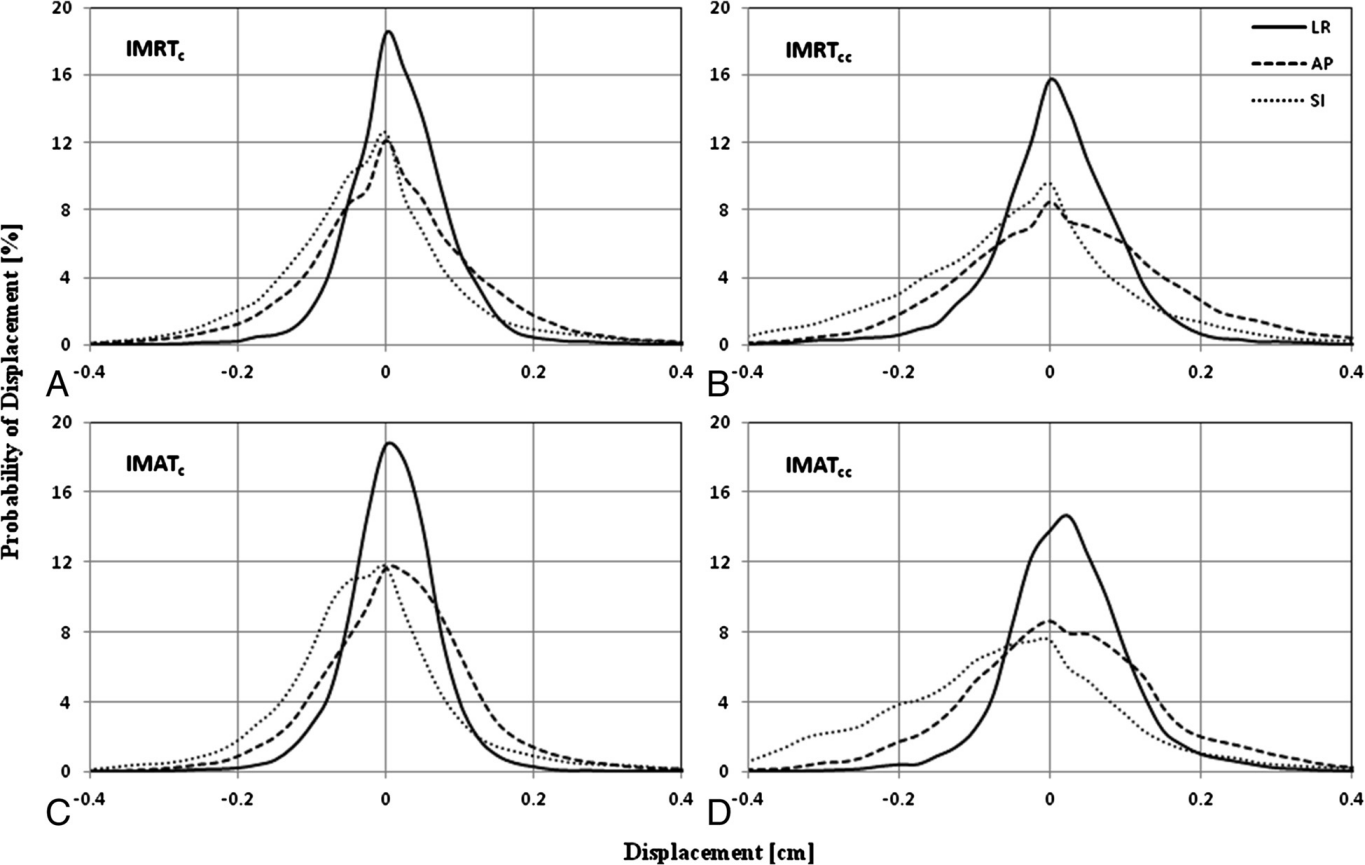
**Histogram analysis showing the probability of displacement of beacon centroid as a function of motion amplitude.** Displacement is measured along the lateral (LR), longitudinal (SI), and frontal (AP) directions for IMRTc **(A)**, IMRTcc **(B)**, IMATc **(C)**, and IMATcc **(D)**.

The probability of isocentre displacement from reference position is presented in Figure 2. For the purpose of histogram analysis, displacement from the reference position was divided into 0.25-mm increments. Movement was most prominent in the SI and AP dimensions in all four tracking categories. Table 1 summarizes the fraction of time the treatment isocentre deviated from its reference position by ≥0 mm, ≥1 mm, ≥2 mm, ≥3 mm, or ≥4 mm in the LR, AP, and SI directions. Longitudinal (SI) motion for treatment categories that included volumetric imaging showed the greatest variability: 17.6% vs. 11.1% of IMRT sessions and 22.2% vs. 9.8% of IMAT sessions had displacement values ≥2 mm. On the other hand, LR motion showed very little variation, again with the most variability observed with the use of volumetric image-guidance. Deviations >5 mm were rare in any dimension secondary to the use of a 4-mm action level corrective intervention protocol. Overall, Table 2 indicates a posterior and inferior prostate drift, with the inferior drift more pronounced than the posterior.

**Table 1:** Isocentre displacement for each treatment category

**Treatment category**	**Displacement [mm]**	**Lateral [%]**		**Vertical [%]**		**Longitudinal [%]**	
		**Left**	**Right**	**Anterior**	**Posterior**	**Superior**	**Inferior**
IMRT_c_	≥0	53.7	46.3	51.2	48.8	35.6	64.4
	≥1	7.1	4.7	16.9	16.2	9.9	24.9
	≥2	0.8	0.6	4.5	4.6	3.9	7.2
	≥3	0.1	0.1	1.1	1.3	1.6	1.7
	≥4	0.0	0.0	0.2	0.4	0.7	0.3
IMRT_cc_	≥0	50.9	49.1	45.8	54.2	36.9	63.1
	≥1	8.7	8.3	18.9	24.0	13.3	29.9
	≥2	1.1	1.6	5.5	8.7	5.5	12.1
	≥3	0.2	0.4	1.4	3.0	2.4	4.5
	≥4	0.0	0.0	0.4	0.9	1.2	1.2
IMAT_c_	≥0	48.2	51.8	47.4	52.6	32.4	67.6
	≥1	3.8	5.6	13.1	14.5	9.0	24.4
	≥2	0.2	0.5	2.4	3.8	3.8	6.0
	≥3	0.0	0.0	0.3	1.1	1.8	1.6
	≥4	0.0	0.0	0.1	0.4	0.9	0.2
IMAT_cc_	≥0	54.6	45.4	47.6	52.4	31.3	68.7
	≥1	9.5	5.4	17.5	20.8	11.0	38.3
	≥2	1.5	0.8	4.5	6.9	4.4	17.8
	≥3	0.2	0.1	1.0	1.8	2.0	6.8
	≥4	0.0	0.0	0.1	0.3	0.9	1.7

**Table 2:** Time to patient support assembly adjustment post initial setup, as well as directional population-based intrafraction motion statistics, as a function of treatment category

**Treatment category**	**Time to patient support assembly adjustment [min]**									**Overall displacement [mean ± std, mm]**			**Group Mean error [M, mm]**			**Systematic error [Σ, mm]**			**R andom error [σ, mm]**		
	**25**^**th **^**Percentile**			**Mean**			**Median**														
	**LR**	**AP**	**SI**	**LR**	**AP**	**SI**	**LR**	**AP**	**SI**	**LR**	**AP**	**SI**	**LR**	**AP**	**SI**	**LR**	**AP**	**SI**	**LR**	**AP**	**SI**
IMRT_c_	--	2.4	2.1	--	3.7	3.4	--	3.4	3.1	0.1 ± 0.6	0.1 ± 1.2	−0.3 ± 1.3	0.05	−0.02	−0.31	0.27	0.5	0.49	0.6	1.09	1.18
IMRT_cc_	6.8	4.5	4.7	6.8	7.8	8.0	6.8	6.5	7.0	0.0 ± 0.8	0.1 ± 1.5	−0.5 ± 1.7	−0.07	0.08	−0.63	0.42	0.65	0.70	0.80	1.32	1.54
IMAT_c_	--	1.9	1.4	--	3.0	2.4	--	2.8	2.3	−0.1 ± 0.5	0.1 ± 1.1	−0.2 ± 1.4	−0.04	0.06	−0.33	0.25	0.57	0.53	0.54	0.90	1.16
IMAT_cc_	10.9	7.4	6.0	13.1	9.9	8.7	13.1	10.1	8.6	0.1 ± 0.8	0.1 ± 1.4	−0.7 ± 1.8	0.05	0.04	−0.77	0.40	0.68	0.72	0.71	1.16	1.54

### Intra-treatment table adjustments

Treatment couch adjustments occurred 601 times out of 4137 treatment sessions. The incidence of required intra-treatment patient support assembly adjustment was as low as 6.6% (IMAT_c_) to 8.3% (IMRT_c_) and as high as 27.2% (IMRT_cc_) to 28.3% (IMAT_cc_). The incidence of treatment couch adjustment was 0.5% in the LR, 30.3% in the AP, and 69.2% in the SI directions, respectively. As expected, the accumulated incidence of table position adjustment was significantly higher for the techniques with longer session duration (IMRT_cc_ and IMAT_cc_) compared with their shorter equivalent (IMRT_c_ and IMAT_c_) in all motion directions. Furthermore, treatment couch adjustments, on average, occurred as early as 2.4 minutes into a tracking session, with the vast majority of table adjustments for the longer treatment sessions occurring onwards of 4.5 minutes (Table 2).

### Prostate displacement as a function of time

Median motion difference amongst treatment categories in the LR direction showed significant difference between IMRT_c_ vs. IMAT_c_ (p=0.0003) and between IMAT_c_ vs. IMAT_cc_ (p=0.0004). In the AP direction, significant difference was observed between IMRT_c_ vs. IMRT_cc_ (p=0.0176) and between IMRT_c_ vs. IMAT_cc_ (p=0.0349). In the SI direction, significant difference was observed between IMRT_c_ vs. IMAT_cc_ (p=0.0003) and between IMAT_c_ vs. IMAT_cc_ (p=0.0021). When adjusted for treatment time, significant difference was observable only between IMRT_c_ and IMAT_c_ (p=0.0024) in the LR direction. Notwithstanding, median motion difference between CBCT and non-CBCT tracking sessions showed strong correlation with time for RMS (t-value=17.29; p<0.0001), SI (t-value=−4.25; p<0.0001) and AP (t-value=2.76; p<0.0066), and weak correlation for LR motion (t-value=1.67; p=0.0971).

**Figure 3:**
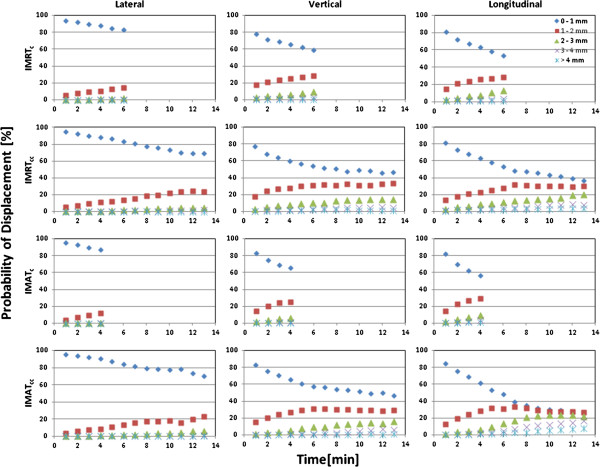
Histogram analysis showing the probability of displacement of treatment isocentre as a function of time for 1-mm displacement intervals.

**Figure 4:**
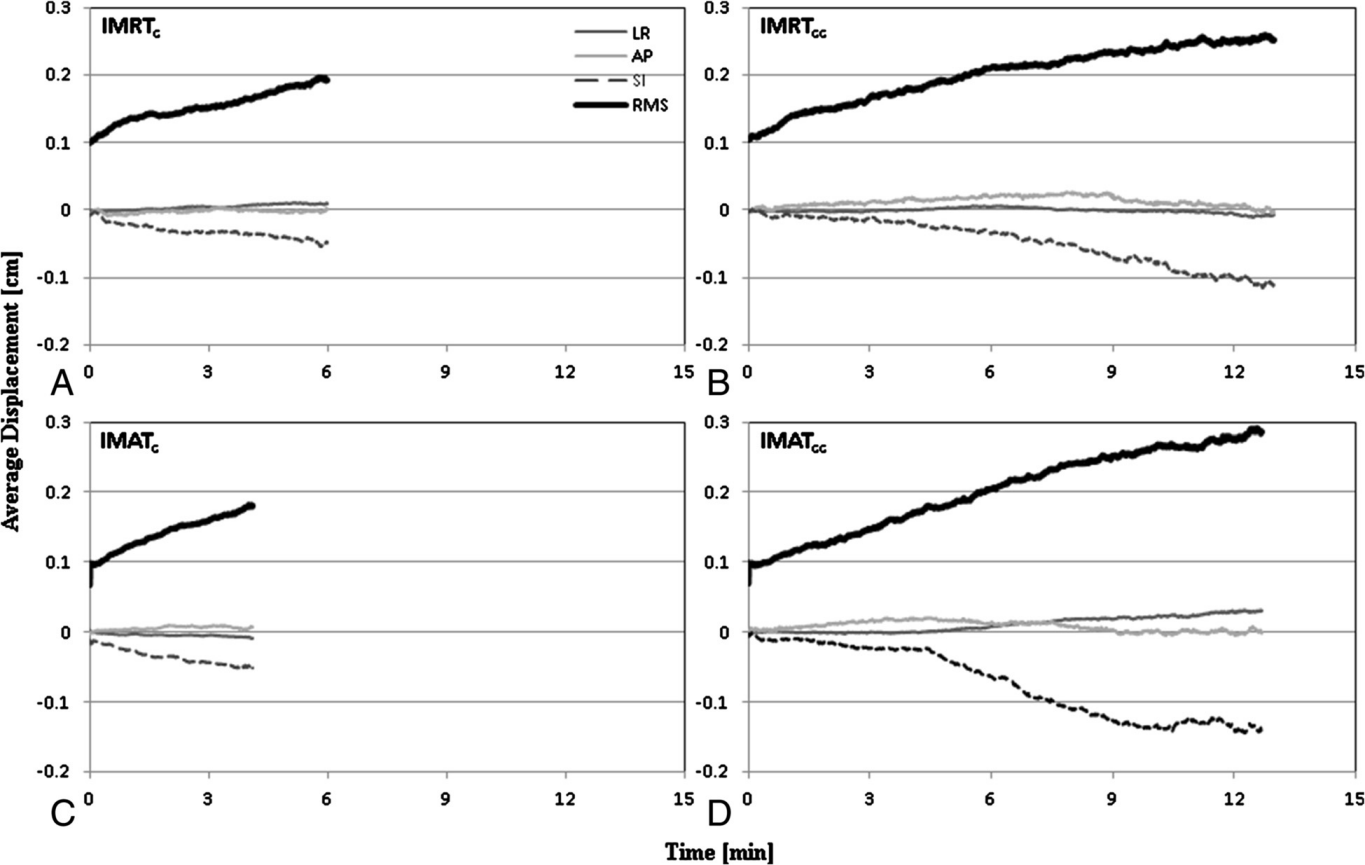
Population-based average treatment isocentre displacement as a function of elapsed time along the lateral (LR), longitudinal (SI), and frontal (AP) directions and as a composite vector for IMRTc (A), IMRTcc (B), IMATc (C), and IMATcc (D).

The mean displacement in the LR and AP directions trended toward 0 mm for the majority of the sampled time points (Figure 3); relating to the fact that deviations were approximately equally distributed in the positive and negative directions. In the LR direction, the standard deviation never exceeded 1 mm; however, the standard deviation was up to 1.5 mm in the AP direction. In the SI direction, the prostate was perceived to drift inferiorly (Figures 2 and 3) and the mean displacement was ≥0.5 mm for techniques with longer treatment session duration (IMAT_cc_ and IMRT_cc_), with a standard deviation exceeding 1.5 mm (for IMAT_cc_ and IMRT_cc_), see Table 2. To further evaluate the effect of time on intrafraction motion, tracking session motion of 1 mm amplitude increments were evaluated over 1-minute intervals (up to the mean tracking time for each corresponding treatment category). When plotted sequentially (Figure 4), an unambiguous trend of increasing motion with time was evident. Table 2 summarizes the results of the observed motion differences between the different treatment categories, indicating that geometric errors increase with increasing tracking time, with the greatest discrepancy observed in the SI followed by the AP direction.

## Discussion

Multiple studies [13,14] have shown that for treatment session time frames shorter than that for image-guided IMRT techniques, intrafraction displacement of the prostate position is insignificant. However, cine magnetic resonance imaging studies [15,16] have shown significant prostate motion at time periods larger than 7 to 9.5 minutes, and have validated the correlation between prostate motion and rectal distension [12,17]. In recent years, high sampling rate (that is, 10–30 Hz) fluoroscopic and electromagnetic systems have been introduced to facilitate on-line setup correction and continuous positional monitoring of implanted markers within target volumes. Consistent with the results of Shelton *et al*. [6], the current study presents an assessment of the temporal dependence of intrafraction motion on the prolongation of treatment time with real-time tumor tracking of electromagnetic transponders. Furthermore, the current study evaluated the impact of the addition of volumetric image-guidance onto IMRT techniques with the use of real-time localization and tracking. The major findings in the current study demonstrate that treatment time reduction for radiotherapy techniques without volumetric image-guidance resulted in significant reductions in motion error; Σ <0.6 mm and σ <1.2 mm compared with Σ ≤0.8 mm and σ <1.6 mm. The current findings are consistent with recent results by Mutanga *et al*. [18] for mean treatments time <5 minutes vs. >11 minutes. The current findings are also generally consistent with prior reports by Kotte *et al*. [10] for short (5–7 minutes) treatment durations.

For each treatment technique evaluated in this study, target displacements progressively increased as a function of elapsed treatment session duration (Figure 2). These findings may be attributable to increased motion around the mean position of the prostate, either internally (due to bladder and/or rectal filling), or externally (due to patient shifts) or related to gradual pelvic floor musculature relaxation [6]. Langen *et al*. showed that mean 3D motion >3 mm progressively increased with treatment time from 2% (during the first minute) to greater than 25% (after 10 minutes) [5]. The latter findings are in concordance with trends observed by Curtis *et al*. [7] and Xie *et al*. [19] for patients with treatment times >5 minutes and with the use of high and low sampling rate motion assessment techniques. These findings are also consistent with results in the current study (see posterior and/or inferior prostate drifts in Table 1). Together, these results are crucial, because intrafraction motion significantly contributes to the systematic and random errors of radiotherapy dose delivery [20] and may result in overdosing to the bladder and/or rectum as well as underdosing to the target volume.

Using a contribution of 2.5 times the systematic error and 0.7 times the random error [20], one can demonstrate that, for the shorter treatment categories (IMRT_c_ and IMAT_c_), the required margins are 1.0–1.1 mm (LR), 2.0–2.1 mm (AP), and 2.0 mm (SI). While these margins are consistent with those reported by Curtis *et al*. [7], Kotte *et al*. [10], and Xie *et al*. [19], they are not in agreement with those reported by Langsenlehner *et al*. [21], in part due to undersampling of motion trajectory with use of very low sampling rate techniques for target motion interrogation in the latter study. Correspondingly, the required margins for the longer tracking treatment categories (IMRT_cc_ and IMAT_cc_) are 1.5–1.6 mm (LR), 2.5 mm (AP), and 2.8–2.9 mm (SI). These are less than the ideal margins proposed by Curtis *et al*. [7] with volumetric imaging every 4 minutes to account for intrafraction motion errors in the absence of positional correction measures based on real-time continuous tracking. It is important to note that the margins reported here are not meant to define an absolute treatment margin due to the fact that only observed target motion is accounted for. Nevertheless, it is evident that comparatively larger margins are required to provide 95% isodose coverage for 90% of the sessions for the longer treatment categories. These large margins can be substantially reduced by continuous monitoring, implementation of an action threshold to reduce the effects of large motions, and keeping treatment sessions short—in this case, shorter that 6 minutes.

### Clinical implications

Although the current results exhibit small intrafraction motion over entire treatment sessions, they also show that even with very accurate online position correction at the start of a treatment session, some positioning uncertainty remains and should be accounted for in margins. Aznar *et al*. [22] reported that when IMAT was used to treat prostate cancer patients, it required less than 2 minutes of beam-on time per treatment. In the current study, within 2 minutes after initial patient setup for daily treatment, the movement of the prostate was limited, though not trivial. Results in the current study suggest treatment session duration should not exceed 4–6 minutes. For treatment duration ≥4-6 minutes, and with the absence of real-time corrective measures for intrafraction motion, it is prudent to incorporate repeat patient localization [7]. Furthermore, in the absence of volumetric image-guidance for repeat patient localization, acquisition of at least the lateral component of an orthogonal image pair needs to be performed, since AP and SI directions are more susceptible to the temporal dependence of intrafraction motion.

Additional clinical strategies to stabilize the prostate gland with the goal of minimizing intrafraction motion include a controlled diet [23] and rectum filling [24-27]. However, these strategies have not garnered widespread clinical application, as their effectiveness when compared with faster treatment techniques, such as IMAT, is yet to be determined.

### Limitations

Table adjustments were made for sustained prostatic displacements that exceeded a predetermined 4 mm threshold. Treatment interventions that occurred during tracking sessions were not retrospectively corrected to void the interventions. As such, the current analysis has the potential to underestimate displacements, particularly those >4 mm. The interpretation of the probability of displacement results, while easily understood from a geometric perspective, may be difficult to implement from a margin formation standpoint, hence, the application of this information to the generation of margins in radiation therapy may require additional evaluation.

## Conclusion

Significant reductions in margins can be achieved through continuous tracking and intrafraction patient repositioning by means of threshold-based intervention. Prolonging treatment duration increases the likelihood of internal organ motion as well as external movement due to patient discomfort, which can impact overall treatment accuracy. Adopting treatment protocols such as IMAT combined with electromagnetic transponder detection and positioning, in addition to continuous monitoring of patient motion, provides an efficient, effective, and accurate delivery of external beam treatments. For treatment durations greater than 4–6 minutes and with no intrafraction motion mitigation protocol in place, patient repositioning is recommended, with the acquisition of at least the lateral component of an orthogonal image pair in the absence of volumetric imaging.

## Abbreviations

σ: Random error; Σ: Systematic error; AP/Y: Anterior-posterior or frontal or vertical; CT: Computed tomography; CBCT: Cone-bean computed tomography; Gy: Gray; IMAT: Intensity-modulated arc therapy; IMATc: IMAT sessions with setup based on electromagnetic transponders only; IMATcc: IMAT sessions with setup based on electromagnetic transponders plus CBCT verification; IMRT: Intensity-modulated radiotherapy; IMRTc: IMRT sessions with setup based on electromagnetic transponders only; IMRTcc: IMRT sessions with setup based on electromagnetic transponders plus CBCT verification; IRQ: Interquartile range; LR/X: Lateral or left-right; M: Population-based mean; RMS: Root-mean-square; SI/Z: Longitudinal or cranio-caudal or superior-inferior; std: Standard deviation.

## Competing interests

The authors declare that they have no competing interests.

## Authors’ contributions

AC participated in the conception and design of the study, performed data analysis, evaluated the results, and drafted the manuscript. AGH participated in the conception and design of the study, performed data analysis, evaluated the results, and drafted the manuscript. SO participated in the conception and design of the study, performed data analysis, evaluated the results, and drafted the manuscript. TSD participated in the design of the study, performed data analysis, evaluated the results, and drafted the manuscript. WMR participated in the design of the study, performed data analysis, evaluated the results, and drafted the manuscript. KER participated in the design of the study, and revised the manuscript. NN participated in the design of the study, and revised the manuscript. TN participated in the statistical analytical assessment of the study data. LZM was responsible for data acquisition, data analysis, evaluation of results, and revision of the manuscript. MF participated in the design of the study, and revised the manuscript. JAT participated in the conception and design of the study, performed data analysis, evaluated the results, and drafted and revised the manuscript. AYH treated all the patients that formed the basis of this study, participated in the design of the study and data analysis, and revised the manuscript. All authors read and approved the final manuscript.

## Pre-publication history

The pre-publication history for this paper can be accessed here:

http://www.biomedcentral.com/1756-6649/13/4/prepub
